# Prevalence of Polyparasitic Infection Among Primary School Children in the Volta Region of Ghana

**DOI:** 10.1093/ofid/ofz153

**Published:** 2019-03-26

**Authors:** Verner N Orish, Jones Ofori-Amoah, Kokou H Amegan-Aho, James Osei-Yeboah, Sylvester Y Lokpo, Emmanuel U Osisiogu, Percival D Agordoh, Festus K Adzaku

**Affiliations:** 1 Department of Microbiology and Immunology, University of Health and Allied Sciences, Ho; 2 Department of Pharmacology, University of Health and Allied Sciences, Ho; 3 Department of Paediatrics, School of Medicine, University of Health and Allied Sciences, Ho; 4 Department of Medical Laboratory Sciences, University of Health and Allied Sciences, Ho; 5 Department of Nutrition and Dietetics, School of Allied Health Sciences, University of Health and Allied Sciences, Ho; 6 School of Basic and Biomedical Sciences, University of Health and Allied Sciences, Ho; 7 Department of Science Laboratory Technology, Wa Polytechnic, Ghana

**Keywords:** polyparasitic infections, helminthes, Plasmodium falciparum, Schistosoma haematobium, school children

## Abstract

**Background:**

Polyparasitic infection is a possibility in areas where parasites are endemic, especially among children. This study looked at the prevalence of polyparasitic infections among children in the Volta Region of Ghana.

**Methods:**

This was a cross-sectional study, among 550 primary school children (aged 6–14 years) in 3 districts in the Volta Region. Questionnaires were administered, and blood, stool, and urine samples were collected. Blood samples were screened for *Plasmodium falciparum* with rapid diagnostic test and microscopy, together with hemoglobin estimation. Stool and urine samples were microscopically examined using wet mount and sedimentation methods to detect intestinal parasites and *Schistosoma haematobium,* respectively. Pearson χ^2^ test was used to evaluate the association between parasitic infections and socioeconomic variables, and multivariate logistic regression to evaluate paired associations among parasites.

**Results:**

The most prominent infection among the children was *P. falciparum* (present in 383 children [69.6%]), followed by *S. haematobium* (57 [10.36%]). There was low prevalence of intestinal protozoa (present in 11 children [2%]), *Ascaris lumbricoides* (7 [1.27%]), and hookworm (5 [0.91%]). A total of 62 children had polyparasitic infection, with *P. falciparum and S. haematobium* having significant paired association (both present in 46 children [74.19%]; adjusted odds ratio, 2.45; *P* = .007).

**Conclusion:**

The prevalence of polyparasitic infection was low in this study, and significant coinfection was seen with *P. falciparum* and *S. haematobium*.

Polyparasitic infection is common among children living in rural areas in developing countries especially in Sub-Saharan Africa. This study showed Plasmodium falciparum and Schistosoma heamatobium as the common polyparastic infection seen among school children in rural Ghana. Parasites are found worldwide [1, 2], but they are replete in developing countries, tropical areas of the world, where, aside from the suitable climate, the poor socioeconomic situation (eg, poor sanitation and inadequate water supply) favors the growth and spread of the organisms causing perennial persistence of diseases in the population [3, 4]. Diseases such as malaria, intestinal parasitosis, and schistosomiasis are very common in many endemic tropical areas of the world [5–7]. These diseases have contributed to significant morbidity and mortality rates in persons living in endemic areas, especially among pregnant women and children [4, 8–10].

Despite the fact that parasites are morphologically diverse, many of them share the same spatial and epidemiological distribution [11, 12]. These overlapping spatial distributions increase the likelihood of multiple parasitic infections in a single susceptible host [1, 13]. Polyparasitic infections occur commonly with endemic parasitic organisms such *Plasmodium falciparum*, intestinal parasites (eg, protozoa and helminths) and *Schistosoma* [11, 14]. The parasite combinations in polyparasitic infections are usually influenced by similar mode of transmission, epidemiological distribution and the immunological modulation and interaction between them [13]. Polyparasitic infections are especially common in rural areas because of poor socioeconomic conditions and among school going children because of behavioral tendencies and underdeveloped immunity [14, 15].

Studies on parasitic diseases very often focus on single parasitic infections, despite overwhelming evidence of coendemicity indicating that polyparasitic infection is probably the norm rather than the exception [1, 12]. This has been demonstrated in several studies done in the Ivory Coast [11, 14, 15], Rwanda [16], Senegal [17], and Chad and Zaire [18]. In Ghana, studies have established the endemicity of malaria [19–21], intestinal parasitosis [22–24], and schistosomiasis [25–27], but only a few have looked at polyparasitic infections in primary school children [28, 29]. With about 40% of Ghanaian population consisting of children <14 years old, and a majority of them living in rural areas [30], it is pertinent that studies be conducted to look at the burden of polyparasitic infection in this vulnerable population. Hence, the current study aimed at estimating the prevalence of polyparasitic infection among primary school children in the Volta Region of Ghana.

## METHODS

### Study Site

This study was undertaken in the southern part of the Volta Region of Ghana, one of the 10 regions in Ghana, bounded by Togo on the east and the Volta Lake and the Eastern Region on the west. It has 25 districts, with the majority of its population living in rural areas. The study was conducted in the Ho municipality, Adaklu and Agotime-Ziope districts.

### Study Population, Design, and Procedure

The study involved 550 children aged 6–14 years in 5 primary schools in these districts and municipality. The 5 schools were purposefully selected from the urban and rural areas of the districts. These included the Freetown primary school in the Ho municipality, the Evangelical Presbyterian primary schools in Afegame and Kpetoe, in the Agotime-Ziope District, and the Dave and Davanu primary schools in the Adaklu District. The study was a cross-sectional study involving administration of questionnaires and the collection of blood, urine, and stool samples from the children. These activities took place from 14 March to 14 April 2016, between 9:30 am and 3 pm each day.

### Sample Size Calculation

With the Cochran formula, 335 was calculated as the minimum sample size for this study at a 95% confidence interval (CI) and 5% margin of error, with a 67.8% prevalence of malaria among school children in Adaklu and Agotime Ziope districts in Volta Region [31].

### Ethical Clearance

Clearance from the Ghana Health Service Ethical Committee (identification no. GHS-ERC: 29/11/15) was obtained for this study. Written informed consent was also obtained from the parents of children who participated in the study, and assent forms were signed by the children before they were enrolled in the study.

### Questionnaire Administration

Demographic and socioeconomic information about the children, including age, parents’ occupations, housing type, and toilet facilities, was obtained from a standardized questionnaire administered in English and the local language (Ewe).

### Sample Collection

First, 3 mL of blood was obtained from the antecubital vein of the right arm and dispensed into a tube containing ethylenediaminetetraacetic acid anticoagulant. Freshly stool and urine samples were also collected from the children in clean containers. All specimen containers were correctly labeled and transported to the laboratory for same-day analysis.

### Laboratory Analysis

Laboratory analysis of the samples was performed as described elsewhere [32, 33]. Briefly, stool samples were analyzed using a wet mount technique. About 2 mg of stool specimen was mixed homogenously with a drop of 0.85% sodium chloride (normal saline) on microscopy slides. The stool smears were subsequently covered with coverslips and examined under a microscope. A trained microscopist examined the smear preparations for the presence of protozoa and ova of helminths. Urine specimens was analyzed using the sedimentation method. About 50 mL of voided urine was obtained from participants. Next, 10 mL of the urine specimen was dispensed into a centrifuge tube and spun for 2 minutes at 2000 rpm. The supernatant was carefully poured out, leaving the sediment, which was resuspended with a few traces of urine left in the tube. The suspension was then placed on microscopy slides and examined under the microscope for the presence of *Schistosoma haematobium* ova. 

Blood samples were used to determine hemoglobin levels and assess for *P. falciparum*. Full blood count analysis was performed using an automated hematology analyzer (Sysmex), and the hemoglobin level was obtained. Anemia was then classified as severe (hemoblogin, <7 g/dL), moderate (7–9.9 g/dL), or mild (<11 g/dL) [34]. Rapid diagnostic test (RDT) kits from Bioline SD (Standard Diagnostics) and Geimsa-stain microscopy techniques were used for to detect *P. falciparum.* For RDTs, a micropipette was used to obtain 5 μL of the stored blood sample and dispensed into the small well of the testing kit with 2 drops of assay buffer subsequently added, and the result was read in 20 minutes. The presence of 2 color bands within the result window indicated a positive result while only 1 band indicates a negative result. 

Giemsa microscopy standard procedure was strictly followed, with both thick and thin films prepared and stained with 10% Giemsa solution for 10 minutes. The thin films, however, were fixed with methyl alcohol for 2–3 minutes before staining. Three microscopists were involved in examining of the slides. Two independently examined the slides initially, and a third microscopist was called on to resolve any discordant results. A slide with asexual forms of the parasite in the blood smear was considered positive.

### Statistical Analyses

A frequency distribution was performed for the variables of age, parents’ occupation, housing type, toilet facility, *P. falciparum*, intestinal protozoa and helminths, *S. haematobium,* and polyparasitic infections. Pearson χ^2^ tests were used to investigate the association between the variables of interest. Based on parasitic infections, the children were divided into 3 groups: those with no infection, those with a single infection, and those with polyparasitic infections. Analysis of variance was used to test for significant differences in the mean hemoglobin levels among the 3 groups of children. Pearson χ^2^ tests were used to investigate the associations between the 3 groups and sociodemographic variables. Multivariate logistic regression was used to estimate paired associations between parasitic infections. Analyses were done with 95% CIs, and differences were considered statistically significant at *P* ≤ .05. All statistical analyses were performed using IBM SPSS Statistics software, version 21.0 (IBM).

## RESULTS

Table 1 highlights the sociodemographic characteristics of the 550 children in this study. The Evangelical Presbyterian primary school in Kpetoe had the most 139 children participating in the study (n = 139), and the Davanu school had the least (n = 79). The study included 249 boys (45.27%) and 301 girls (54.73), no significant difference in the sex distribution among the 5 primary schools (*P* = .36). Trading and farming were the predominant occupations for the children’s fathers (trading, 254 fathers [46.18%]; farming, 170 [30.91%]) and mothers (334 [60.73%] and 156 [28.36%] mothers, respectively). The occupation of farming was significantly predominant among parents of children from the Afegame (father, 45 fathers [26.47%] and 55 mothers [35.26%]; *P* < .001) and Davanu (56 [32.94%] and 47 [30.13%], respectively; *P* < .001) schools. Open or bush defecation was also more common among children from the Afegame school (17 [34.69%]; *P* < .001)

**Table 1.** Sociodemographic Characteristics of Children Stratified by Primary Schools

Characteristic	Children, No. (%) by School^a^						
	Dave (n = 84)	Davanu (n = 79)	Freetown (n = 125)	Afegame (n = 123)	Kpetoe (n = 139)	Total (N = 550)	*P* Value^b^
Age, mean (SD), y	10.76 (2.85)	11.32 (3.35)^c^	10.93 (2.68)	10.08 (2.32)	11.46 (2.14)^c^	10.90 (2.66)	<.001
Sex							
Male	37 (14.86)	38 (15.26)	51 (20.48)	64 (25.70)	59 (23.70)	249 (45.27)	.36
Female	47 (15.61)	41 (13.62	74 (24.59)	59 (19.60)	80 (26.58)	301 (54.73)	
Father’s job							
Trader	43 (16.93)	20 (7.87)	61 (24.02)	56 (22.05)	74 (29.13)	254 (46.18)	<.001
Farmer	22 (12.94)	56 (32.94)	19 (11.18)	45 (26.47)	28 (16.47)	170 (30.91)	
Civil servant	19 (18.45)	1 (0.97)	38 (36.89)	16 (15.53)	29 (28.16)	103 (18.73)	
Unemployed	0 (0.00)	2 (8.70)	7 (30.43)	6 (26.09)	8 (34.78)	23 (4.18)	
Mother’s job							
Trader	60 (17.96)	26 (7.78)	92 (27.55)	57 (17.07)	99 (29.64)	334 (60.73)	<.001
Farmer	18 (11.54)	47 (30.13)	14 (8.97)	55 (35.26)	22 (14.10)	156 (28.36)	
Civil servant	3 (8.83)	1 (2.94)	13 (38.24)	6 (17.65)	11 (32.35)	34 (6.18)	
Unemployed	3 (11.54)	5 (19.23)	6 (23.08)	5 (19.23)	7 (26.92)	26 (4.73)	
House type							
Family house	41 (20.92)	17 (8.67)	39 (19.90)	56 (28.57)	43 (21.94)	196 (35.64)	<.001
Single room	15 (9.09)	23 (13.94)	40 (24.24)	37 (22.42)	50 (30.31)	165 (30.00)	
Semidetached	28 (14.82)	39 (20.63)	46 (24.34)	30 (15.87)	46 (24.34)	189 (34.36)	
Toilet facility							
Within house	52 (15.25)	37 (10.85)	101 (29.62)	60 (17.60)	91 (26.68)	341 (62.00)	<.001
Community toilet	26 (16.25)	34 (21.25)	18 (11.25)	46 (28.75)	36 (22.50)	160 (29.09)	
Open bush toilet	6 (12.25)	8 (16.33)	6 (12.25)	17 (34.69)	12 (24.48)	49 (8.91)	

Abbreviation: SD, standard deviation.

^a^Data represent no. (%) of children unless otherwise specified.

^b^Significant at *P* ≤ .05.

^c^Significantly different from the Afegame school.

**Figure 1.**
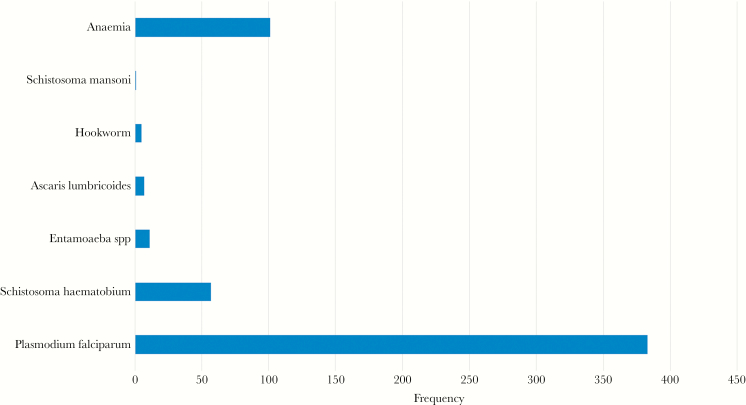
Prevalence of parasitic infection and anemia among the children.

Figure 1 shows the prevalence of infection and anemia seen in the children in this study. *P. falciparum* was the predominant infection, with 383 children (69.64%) testing positive with either RDTs or microscopy. Fifty-seven children (10.36%) were infected with *S. haematobium*. Only 1.27% of the children in this study (7 of 550) were infected with *Ascaris lumbricoides*, 0.91% with hookworm (5 of 550), and 2% with intestinal protozoa (ie, *Entamoeba* spp.) (11 of 550). Only 1 child had *Schistosoma mansoni* infection. Anemia was observed in 102 children (18.54%).

Table 2 shows the distribution of infections and anemia among the children in the 5 schools. The Afegame school had the highest prevalence of *P. falciparum* (107 children [86.99%]) and *S. haematobium (*34 [27.64%]) infections (both *P* < .001), and the highest prevalence of children with anemia (36 [29.27%]; *P* = .003).

**Table 2.** Distribution of Parasitic Infections and Anemia Among School Children in the Ho Municipality

Infections and Anemia	Children by School, No. (%)					*P* Value^a^
	Dave	Davanu	Freetown	Afegame	Kpetoe	
*Plasmodium falciparum*	57 (67.86)	61 (77.22)	73 (58.40)	107 (86.99)	85 (61.15)	<.001
*Schistosoma haematobium*	5 (5.95)	14 (17.72)	4 (3.20)	34 (27.64)	0 (0.00)	<.001
*Entamoeba* spp.	8 (9.52)	0 (0.00)	1 (0.80)	1 (0.81)	1 (0.71)	<.001
*Ascaris lumbricoides*	4 (4.76)	0 (0.00)	1 (0.80)	1 (0.81)	0 (0.00)	<.001
Hookworm	2 (2.38)	0 (0.00)	3 (2.40)	1 (0.81)	1 (0.71)	<.001
Anemia	15 (17.86)	15 (18.99)	14 (11.20)	36 (29.27)	22 (15.83)	.003

^a^Significant at *P* ≤. 05.

Table 3 shows the 3 groups of primary school children in terms of their infection status (no infection, single and polyparasitic infection). Only 151 children (27.46%) were free of parasitic infection in this study, and 336 (61.09%) had a single infection, either *P. falciparum* (320 children [95.24%]), *S. haematobium* (10 [2.98%]), or intestinal parasites (6 [1.78%]). The predominant polyparasitic infection was double infection (62 children [11.27%]), coinfection with *P. falciparum* and either *S. haematobium* (46 children [74.19%]) or intestinal parasites (16 [25.81%]). Only 1 child had infections of *S. mansoni, S. haematobium,* intestinal protozoa, and *P. falciparum*. Although more boys (28 children [60.87%]) were coinfected with *P. falciparum* and *S. haematobium* and more girls (180 [56.25%]) with a single infection of *P. falciparum,* these findings were not significant (*P* = .17). Coinfection was significantly more common among children from the Afegame school (28 [60.87%]; *P* < .001). There was a significant paired association with *P. falciparum* and *S. haematobium* infection; of 57 children who were *S. haematobium* positive, 47 were *P. falciparum* positive, and 10 were *P. falciparum* negative (*P* < .004) (adjusted odds ratio, 2.45 [95% CI, 1.32–4.42]; *P* = .007). 

**Table 3.** Parasitic Infection Pattern Among School Children in the Ho Municipality

Parameter	No Infection	Children, No. (%)^a^					*P* Value^b^
		Monoinfection			Polyparasitic Infection		
		*Plasmodium*	*Schistosoma*	Intestinal Parasitosis	Plasmodium +*Schistosoma haematobium*	*Plasmodium* + Intestinal Parasite	
All children^c^	151 (27.46)	320 (58.18)	10 (1.82)	6 (1.09)	46 (8.36)	16 (2.91)	
Age, mean (SD), y	11.29 (2.73)	10.7 (2.37)	10.5 (2.12)	10.13 (2.85)	10.76 (2.05)	10.06 (2.15)	.32
Sex							
Male	60 (39.74)	140 (43.75)	4 (40.00)	2 (33.33)	28 (60.87)	9 (56.25)	.17
Female	91 (60.26)	180 (56.25)	6 (60.00)	4 (66.67)	18 (39.13)	7 (43.75)	
School							
Dave	23 (15.23)	42 (13.12)	1 (10.00)	3 (50.00)	3 (6.52)	11 (68.75)	<.001
Freetown	50 (33.11)	67 (20.94)	0 (0.00)	2 (33.33)	4 (8.70)	2 (12.5)	
Davanu	14 (9.27)	51 (15.94)	4 (40.00)	0 (0.00)	10 (21.74)	0 (0.00)	
Afegame	11 (7.28)	76 (23.75)	5 (50.00)	1 (16.67)	28 (60.87)	2 (12.50)	
Kpetoe	53 ( (35.09)	84 (26.25)	0 (0.00)	0 (0.00)	0 (0.00)	1 (6.25)	
Anemia	22 (14.91)	60 (18.75)	0 (0.00)	0 (0.00)	17 (36.96)	3 (18.75)	.051
Hemoglobin, g/dL	12.05	11.87	11.98	11.89	12.41	11.7	.50

Abbreviation: SD, standard deviation.

^a^Data represent no. (%) of children unless otherwise specified.

^b^Significant at *P* ≤ .05.

^**c**^Percentages calculated with the total sample of 550 as the denominator.

## Discussion

The current study examined the burden of polyparasitic infection among school children in 5 primary schools in 2 districts and 1 municipality in the Volta Region of Ghana. This is among the few studies that have investigated polyparasitic infection in children in the region.

*P. falciparum* infection was the most prominent parasitic infection in this study, with most (69.6%) of the children testing positive with either RDT or microscopy. This high prevalence is not uncommon in this part of the region where the present study was conducted; a previous study recorded a prevalence of 67.8% among children aged 6–12 years [31], although a much lower prevalence was also reported among school-age children in another district in the region [29]. All children with *P. falciparum* infection in this study were asymptomatic. This is a common presentation of infection among this age group in malaria endemic areas [35]. As immunity gradually improves in older children, there is concomitant decline of clinical malaria and an increase in asymptomatic presentations [35].

There was a low level of intestinal parasitic and *S. haematobium* infections in this study. The active periodic deworming exercise among school children in the region, targeting intestinal helminths and *S. haematobium,* might have been responsible for these low levels [36]. This low prevalence among school children has also been reported in another study in the region [29], although it is important to note that the wet mount technique used in this study may have contributed to the low prevalence, as it is judged to be less sensitive than the Kato-Katz and formol-ether concentration methods [37]. However, it is important to mention that even the previous study done in the region, which used the concentration techniques, also recorded no cases of intestinal helminth infection [29].

Polyparasitic infection was seen in only 11.5% of the children in this study. This prevalence is low compared with findings of studies from other parts of Africa [11, 14, 15, 17, 18]. The predominant and significant polyparasitic infection seen was *P. falciparum* and *S. haematobium* coinfection. This predominant coinfection has been reported in other studies [14, 17]. This is not really surprising, because in the current findings and those from some others, *P. falciparum* and *S. haematobium* infections are the 2 most prominent infections among school children in endemic areas [14, 17]. 

Children from the Afegame school were significantly more likely than those from other schools to be coinfected with *P. falciparum* and *S. haematobium*. Again, this was expected, because the highest prevalences of both infections were noted among the children in this school. The Afegame school is located in a typical rural, farming and riparian community with poor socioeconomic and environmental conditions that could aid the spread of both *P. falciparum and S. haematobium* [20, 29]. Moreover, studies have reported polyparasitic infections occurring more in rural parts of Africa [11, 14, 15, 17, 18]. Coinfected children were also noted to have the highest prevalence of anemia. This finding could support the notion of a probable positive interaction, with synergistic and/or additive effects of both *P. falciparum and S. haematobium*, because both are known to cause anemia [38, 39]. This finding, however, is in contrast with findings from some other studies that reported a decreased odds of anemia among children coinfected with *P. falciparum and S. haematobium* [14, 17].

An important limitation of the current study is the single stool and urine samples obtained from study participants, which might have contributed to low levels of intestinal parasites and *S. haematobium*. Multiple or serial stool and urine sample collection improves the chances of finding parasites in samples from an infected person [29, 40]. However, the low level of intestinal parasites from this study corroborated the finding of a previous study in the region [29]. This suggests that the low level of intestinal parasites among school children might reflect the actual situation in the region at the time of this study, which could be attributed to the periodic deworming exercise.

In conclusion, the current study shows that the burden of polyparasitic infection among primary school children was relatively low, with high prevalence of single infection of *P. falciparum* followed by *S. haematobium* infection. There was predominant and significant coinfection of *P*. *falciparum* and S. *haematobium*, prominent among children in the rural area of the Afegame primary school, and this coinfection is associated with a high prevalence of anemia. More studies are needed to further evaluate the burden of polyparasitic infection, the interactions that exist between them, and their impact on morbidity and mortality rates among children in the region.
